# Stepped care to optimize pre-exposure prophylaxis (PrEP) effectiveness in pregnant and postpartum women (SCOPE-PP) in South Africa: a randomized control trial

**DOI:** 10.1186/s12889-022-13652-5

**Published:** 2022-07-07

**Authors:** Dvora Leah Joseph Davey, Kathryn Dovel, Susan Cleary, Nehaa Khadka, Nyiko Mashele, Miriam Silliman, Rufaro Mvududu, Dorothy C. Nyemba, Thomas J. Coates, Landon Myer

**Affiliations:** 1 Department of Epidemiology, Fielding School of Public Health, University of California Los Angeles, Los Angeles, CA USA; 2 Division of Epidemiology and Biostatistics, School of Public Health, University of Cape Town, Cape Town, South Africa; 3 Division of Infectious Diseases, David Geffen School of Medicine, University of California Los Angeles, 0833 Le Conte Ave, Los Angeles, CA 90095 USA; 4 Division of Health Economics, School of Public Health, University of Cape Town, Cape Town, South Africa

**Keywords:** Pre-exposure prophylaxis, PrEP, PMTCT, Pregnant, Breastfeeding, South Africa, Persistence, Persistence, Protocol, Randomized control trial, Economic evaluation

## Abstract

**Background:**

HIV incidence among pregnant and postpartum women remains high in South Africa. Pre-exposure prophylaxis (PrEP) use remains suboptimal in this population, particularly during the postpartum period when women’s engagement with routine clinic visits outside PrEP decreases. Key barriers to sustained PrEP use include the need for ongoing contact with the health facility and suboptimal counseling around effective PrEP use.

**Methods:**

Stepped Care to Optimize PrEP Effectiveness in Pregnant and Postpartum women (SCOPE-PP), is a two-stepped unblinded, individually randomized controlled trial (RCT) that aims to optimize peripartum and postpartum PrEP use by providing a stepped package of evidence-based interventions. We will enroll 650 pregnant women (> 25 weeks pregnant) who access PrEP at a busy antenatal clinic in Cape Town at the time of recruitment and follow them for 15 months. We will enroll and individually randomize pregnant women > 16 years who are not living with HIV who are either on PrEP or interested in starting PrEP during pregnancy. In step 1, we will evaluate the impact of enhanced adherence counselling and biofeedback (using urine tenofovir tests for biofeedback) and rapid PrEP collection (to reduce time required) on PrEP use in early peripartum compared to standard of care (SOC) (*n* = 325 per arm). The primary outcome is PrEP persistence per urine tenofovir levels and dried blood spots of tenofovir diphosphate (TFV-DP) after 6-months. The second step will enroll and individually randomize participants from Step 1 who discontinue taking PrEP or have poor persistence in Step 1 but want to continue PrEP. Step 2 will test the impact of enhanced counseling and biofeedback plus rapid PrEP collection compared to community PrEP delivery with HIV self-testing on PrEP use (n = up to 325 postpartum women). The primary outcome is PrEP continuation and persistence 6-months following second randomization (~ 9-months postpartum). Finally, we will estimate the cost effectiveness of SCOPE-PP vs. SOC per primary outcomes and disability-adjusted life-years (DALYs) averted in both Step 1 and 2 using micro-costing with trial- and model-based economic evaluation.

**Discussion:**

This study will provide novel insights into optimal strategies for delivering PrEP to peripartum and postpartum women in this high-incidence setting.

**Trial registration:**

NCT05322629: Date of registration: April 12, 2022.

## Background

Women in sub-Saharan Africa face high HIV acquisition risk during pregnancy and breastfeeding with HIV incidence doubling during pregnancy and the postpartum period compared with non-pregnant/breastfeeding women [1–3]. Seroconversion during pregnancy or breastfeeding substantially increases the risk of vertical transmission, and accounts for nearly one-third of all infant HIV infections [4, 5]. As fertility rates in the region remain high [6], high HIV risk during pregnancy and postpartum translates to a substantial cumulative period of risk for women spanning over decades of women’s lives, underscoring the urgent need for prevention interventions tailored to at-risk pregnant and postpartum women. While the elimination of mother-to-child transmission (eMTCT) services have expanded rapidly in the region [7–9], few prevention interventions exist for most pregnant women who initially test HIV-negative. This is a major missed opportunity that has implications for women, their partners, and infants.

The World Health Organization (WHO) recommends offering pre-exposure prophylaxis (PrEP) to pregnant and postpartum women at risk for HIV acquisition as a female-controlled prevention strategy [10–12]. The South African National PrEP guidelines were updated in 2020 to include offering PrEP to PBFW not living with HIV [13]. However, current oral PrEP (TDF-FDC) must be taken daily for it to be effective. PrEP persistence in women has been low in South Africa and surrounding countries [14–18]. Our PrEP in pregnant and postpartum (PrEP-PP) study was one of the first studies to integrate PrEP into government maternal and child health care services in South Africa. Preliminary findings from the study show high levels of PrEP initiation (> 85% of eligible women), but low levels of continuation on PrEP (< 60% of women at 6 months postpartum) [19]. Innovative PrEP optimization strategies are needed to reach PBFW at risk of HIV acquisition, particularly postpartum.

Barriers to optimal PrEP use among PBFW span across health facility-, intrapersonal-, and interpersonal-levels. In surveys and in-depth interviews with women on PrEP, our team found that many women underestimated their partner’s risk of living with HIV [20–22]. Women often reported needing permission from their partners to use PrEP and feared disclosing to their partners that they are taking PrEP. Noticeable drop-offs in clinic visits for PrEP collection and PrEP persistence among postpartum women were largely contributed to decreased clinic attendance postpartum (as women no longer attended antenatal care (ANC) services) and periods of postpartum abstinence where they had low risk of HIV acquisition and therefore stopped taking PrEP [23–25]. For postpartum women, PrEP consultations may be the only reason to visit the facility, adding substantial time and travel burden. Finally, standard PrEP counseling is based on self-reported persistence, which may over report drug persistence and therefore miss opportunities for in-depth, tailored persistence counseling that meet individual clients’ needs [26].

Stepped care to optimize PrEP effectiveness in pregnant and postpartum women (SCOPE-PP) is a two stepd, unblinded, individually randomized controlled trial (RCT) that aims to optimize postpartum PrEP use by providing a stepped package of evidence-based interventions. The intervention includes two steps implimented across two steps of the RCT: Step 1 intervention arm includes the offer of enhanced adherence counselling through biofeedback of tenofovir levels following a rapid urine tests and rapid PrEP collection (following HIV self-testing, [HIVST]); Step 2 women with poor PrEP continuation or persistence in Step 1 to either, (a) rapid PrEP (following HIVST) and the choice of commuinty PrEP pick-up points, or (b) biofeedback adherence counseling in the clinic. Intervention arms will be compared to the standard of care PrEP delivery per the South African National Department of Health guidelines [27]. As part of the study we will estimate the cost effectiveness of SCOPE-PP vs. SOC per primary outcomes and disability-adjusted life-years (DALYs) averted in both Step 1 and 2 using micro-costing with trial- and model-based economic evaluation. By identifying optimal strategies for delivering PrEP to PBFW, this study can improve upon exisiting PrEP strategies to decrease HIV acquisition and vertical transmission in the region.

## Methods

### Aims

The aims of the study are to:

- *Aim 1*: Evaluate the impact of SCOPE-PP interventions on PrEP persistence in pregnancy and early postpartum (peripartum) (Step 1) and postpartum women (Step 2)
- Step 1 (randomize 650 pregnant women actively taking PrEP at the time of enrollment):
  - Intervention: Enhanced adherence counseling through biofeedback and rapid PrEP collection following HIVST
  - Primary Outcome 1: PrEP continuation and persistence per urine tenofovir (TFV) at 6-months after randomization (verified post hoc via dried blood spots [DBS] analysis of tenofovir diphosphate [TFV-DP]).
  - Hypothesis 1: Enhanced adherence counseling and rapid PrEP collection will improve PrEP continuation and persistence by > 15%.
- Step 2: We will randomize ~ 325 postpartum women from Step 1 who disengaged from PrEP during the first 6-months post enrollment and desire to stay on PrEP to:
  - Intervention 1: Rapid PrEP collection following HIVST and differentiated model of community-based PrEP delivery.
  - Intervention 2: Enhanced adherence counseling through biofeedback in the clinic and rapid PrEP collection following HIVST
  - Primary Outcome 1: PrEP continuation and persistence per urine TFV at 6-months after randomization in Step 2 (verified post hoc via DBS analysis of TFV-DP).
  - Hypothesis 2: Among postpartum women who struggle to engage with PrEP within the first 6-months postpartum, differentiated PrEP delivery (in community pick up points) will improve PrEP continuation and persistence by > 15% compared to standard-of-care (intensive counseling and facility PrEP delivery) at 12-months postpartum.
- *Aim 2****:*** Evaluate the cost effectiveness and equity impact of SCOPE-PP vs. standard of care. Within a trial-based cost-effectiveness analysis, we will estimate provider costs and primary outcomes of SCOPE-PP in terms of PrEP continuation and persistence per urine tenofovir at 6-months and 6-months after randomization in Step 2. Thereafter, in a model-based economic evaluation that integrates HIV-infections averted, we will estimate HIV-treatment cost offsets in order to estimate lifetime costs and DALYs averted. The resulting incremental cost per DALY averted will be compared to a South African cost-effectiveness threshold to assess value for money [28].

### Trial design

We will conduct a two-stepped (i.e., two randomization points), unblinded individually randomized controlled trial (RCT) that aims to optimize postpartum PrEP use by providing a stepped package of evidence-based interventions. The RCT takes a pragmatic approach and utilizes routine providers for intervention delivery to promote fidelity for real-world settings.

### Setting

This study will be implemented in the Gugulethu Midwife Obstetrics Unit (MOU) in Cape Town. Gugulethu’s population of 300,000 is predominantly of low socioeconomic status (SES). This township has 48% unemployment rate and 64% of the adult population live on ~$35 USD per month. The population uses public-sector health services that are provided free at the point-of-use. In 2018, the HIV prevalence among women attending ANC services provided by the MOU was 27% with > 80% of women breastfeeding. We will build on the existing infrastructure at each clinic and train routine providers on the PrEP intervention to be provided within clinical visits. Additional study visits will be provided in a research site behind the clinic by trained UCT qualitative and quantitative interviewers who speak the local language, isiXhosa.

### Conceptual framework

**Fig. 1:**
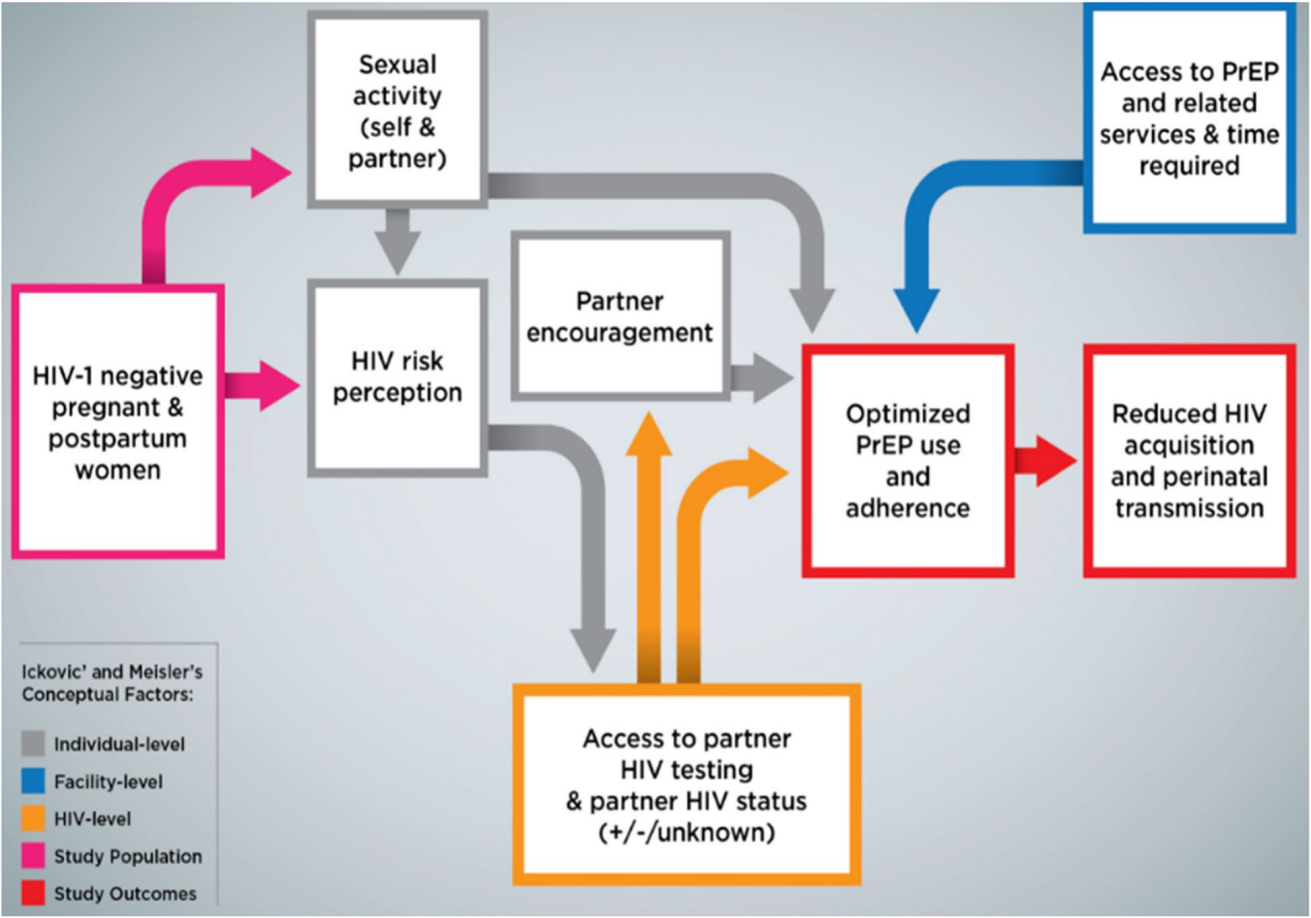
Conceptual framework for SCOPE-PP study

We adapted Ickovics’ and Meisler’s conceptual framework with factors affecting persistence in HIV treatment [29] to place our study and its outcomes into a broader persistence context (Fig. 1). The conceptual factors are categorized by 1) facility-level, individual-level, 2) and 3) HIV-level from the participants’ perspective which were key factors for pregnant and postpartum persistence in our PrEP-PP study [22, 23].

### Ethical review

The study protocol, informed consent form, all data collection tools, and other requested documents have been reviewed and approved by the University of Cape Town Faculty of Health Sciences Human Research Ethics Committee (UCT-HREC). University of California Los Angeles IRB has provided ethical reliance on UCT-HREC. Participants will be reimbursed for their time and transportation (15USD/R120/per study visit) to the clinic for each study visits (no reimbursement for travel undergone to collect PrEP in the clinic or community in Step 2).

#### Step 1: EARLY-PREP IN PERIPARTUM

##### Recruitment and enrolment

Pregnant women who are on PrEP or initiate PrEP at baseline visit will be recruited and screened for eligibility during their routine ANC services at gestational > 25 weeks using a set script for screening and a brief description of the study. We have recruited over 1300 pregnant women in our prior study in 18 months and are confident that we can continue to recruit from this facility. We will obtain written informed consent immediately following screening.

The inclusion criteria for Step 1 include:

1. Age ≥ 16 years (16- and 17-year-old pregnant adolescent girls will provide unassisted consent)
2. > 25 weeks pregnant
3. Plans to deliver at the study facility (or nearby hospital)
4. Documented HIV-negative (per national protocol for routine ANC),
5. Lives within 20 km of the study facility
6. On PrEP prior to study or interested in starting PrEP in study visit
7. Willing and able to consent to study participation.

Individuals not meeting the above criteria will be excluded and referred to SOC PrEP services.

##### Randomization (Fig. 2)

For Step 1 (pregnant women on PrEP or wanting to start PrEP at the time of enrollment), individual women will be randomized using a 1:1 ratio to either:

1. Standard of Care (SOC) or
2. Enhanced adherence counselling through biofeedback and rapid PrEP collection.

**Fig. 2:**
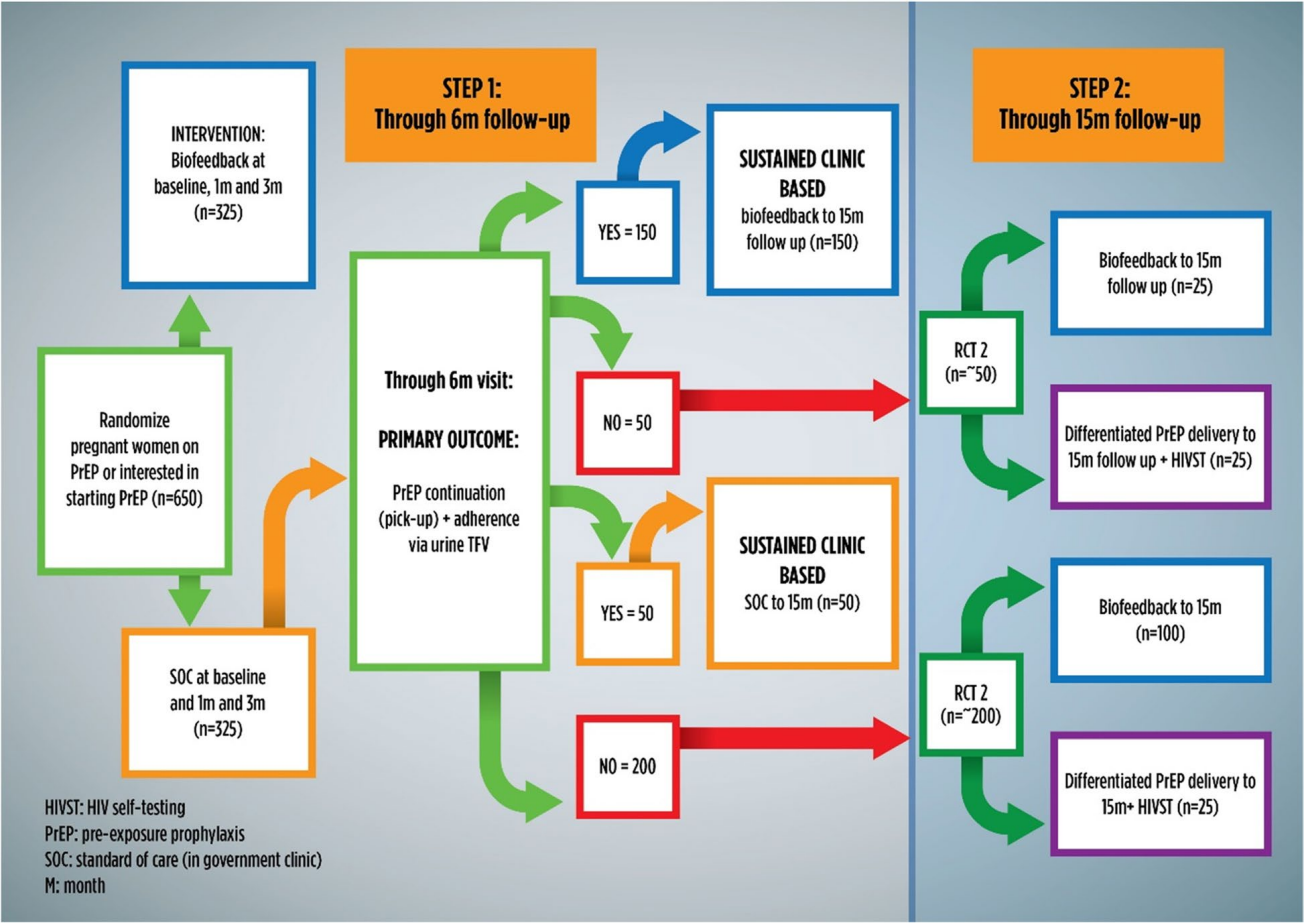
SCOPE-PP Study Design: Step 1 and Step 2

After enrolling in the trial and completing a baseline survey, PBFW will be assigned immediately to a study ID based on the randomization list. Study ID’s will be linked with the pre-assigned blocked randomization by the study data manager and pre-loaded into the tablet device but will be unknown to the study staff until survey and randomization modules are completed and saved, ensuring randomization cannot be manipulated by the study staff. Once finalized, the randomization results will appear on the tablet device as a picture on a pre-programmed tablet, and will be shown to the participant to maximize transparency and study buy-in.

##### Standard of care

Women randomized to the SOC arm (*n* = 325) will receive facility-based PrEP, blood-based, provider administered HIV testing and PrEP counseling monthly during pregnancy and quarterly during postpartum. Women will also receive HIV self-screening kits (HIVST) during each facility visit to take home to their sexual partners, per South African HIV testing guidelines. PrEP prescriptions and HIV testing will be provided according to the national PrEP guidelines in 3 monthly intervals [13].

##### Intervention (step 1; Table 1)

Women randomized to the intervention arm (*n* = 325) will receive SOC services (described above) plus bio-feedback persistence counseling based on urine lateral flow assays of TFV to measure recently daily adherence including rapid PrEP delivery following HIVST. Intervention steps are detailed below.

- **Real-time novel immunoassay using urine that measures TFV and enhanced bio-feedback counseling (10 minutes):** This immunoassay (UrSure, OraSure Technologies, Inc.) is sensitive (100%) and specific (97%) when compared to plasma TFV levels [30] and will be used to identify women who may need additional counseling or differentiated PrEP delivery. The novel urine assay shows TFV concentrations if TDF is taken in the past 48 hours thereby enabling counselors to provide feedback on persistence levels, immediately at point of care. Prior studies, including our own pilot in postpartum women [21] have demonstrated the efficacy, feasibility, and acceptability of urine TFV testing.
- **Enhanced counseling (15 minutes):** includes:

1. Feedback of urine TFV test results to encourage continued persistence or the development of a plan for how improve daily use, especially prior to and during periods of sexual activity (prevention effective adherence)
2. Counseling on the importance of knowing the partners’ serostatus (and possibility of serodiscordant results), and
3. All other SOC counseling topics, including highlighting the risk of seroconversion and the importance of PrEP persistence and regular quarterly HIV testing while on PrEP.

- **Rapid PrEP collection (3 minutes):** At study enrollment clients will be given HIV self-test kits (HIVST) to be used for themselves, in addition to their partners if they do not know their serostatus. Prior to the repeat PrEP clinical visit (every 3 months), participants can personally use a HIVST kit and share a photograph of results or actual used HIVST kit to speed up the PrEP collection process. Those with a non-reactive HIVST kit will forego additional HIV testing at that visit (cutting at least 15 minutes from PrEP collection procedures). Women can collect a pre-packaged 3-month supply of PrEP following adherence counseling and monitoring of side effects.

**Table 1:** Study and clinic visits by intervention and standard of care arms in Step 1 of RCT in SCOPE-PP study, Cape Town, South Africa

RCT Step 1: EARLY PrEP-P	SOC (***n*** = 325)	Intervention (***n*** = 325)	Steps and measurement
**Randomization of pregnant women on PrEP (= > 25 weeks gestation)**			
**Baseline Study visit**	In clinic: PrEP offer, standard counseling	In clinic: PrEP offer, standard counseling	In study site (behind clinic): • Consent form • Survey • Reimbursement for time
**1 month clinic visit**	In clinic: PrEP refill and standard counseling, urine testing (no feedback)	In clinic: PrEP refill with urine testing and feedback on PrEP levels in urine	Standard monitoring No reimbursement for time
**3 month Study visit**	In clinic: PrEP refill and standard counseling, urine testing (no feedback) With standard HIV testing/HIVST	In clinic: PrEP refill with urine testing and feedback on PrEP levels in urine With standard HIV testing/HIVST	In study site: • Blood collection • Survey • Reimbursement for time
**6 month** **Study visit**	In clinic: PrEP refill and standard counseling, urine testing (no feedback) With standard HIV testing/HIVST	In clinic: PrEP refill with urine testing and feedback on PrEP levels in urine With standard HIV testing/HIVST	In study site: • Blood collection • Survey • Reimbursement for time
**If participant continues on PrEP and adherent based on urine testing, can continue in study according to following visits. If not adherent or discontinues, can participate in RCT Step 2 (LATE-PREP-P).**			
**9 month clinic visit**	In clinic: PrEP refill and standard counseling, urine testing (no feedback) With standard HIV testing/HIVST	In clinic: PrEP refill with urine testing and feedback on PrEP levels in urine With standard HIV testing/HIVST	Standard monitoring No reimbursement for time
**12 month Clinic visit**	In clinic: PrEP refill and standard counseling, urine testing (no feedback) With standard HIV testing/HIVST	In clinic: PrEP refill with urine testing and feedback on PrEP levels in urine With standard HIV testing/HIVST	Standard monitoring No reimbursement for time
**15 month Study visit**	In clinic: PrEP refill and standard counseling, urine testing (no feedback) With standard HIV testing/HIVST	In clinic: PrEP refill with urine testing and feedback on PrEP levels in urine With standard HIV testing/HIVST	In study site: • Blood collection • Survey • Reimbursement for time
**End of study and referral for ongoing PrEP care in clinic**			

##### In case of a seroconversion

In both arms, women on PrEP with a reactive result will be followed up by study staff and counseled to stop taking PrEP immediately until they confirm their serostatus and initiate ART. If they are unable to attend the facility, study staff will do a home visit to follow up.

### Data collection

#### Baseline survey

Participants will complete a baseline survey immediately following enrollment. The baseline survey will collect key data on socio-demographic information, partner and pregnancy history, HIV risk (including sexual activity, partner HIV status and risk perception), prior use of PrEP and attitudes about PrEP, and information on IPV and substance use. All surveys will be conducted in the local language by trained study staff using electronic tablets with REDCap software. Participants who report substance use, violence and/or IPV will be referred to a local NGO for social services and counseling in addition to study activities.

#### Follow-up surveys

Participants will complete 4 follow-up surveys every 3 months (study follow-up visits separate from clinical PrEP visits). Follow-up surveys will document any changes in HIV risk, sexual activity, and PrEP persistence, including pill count. Self-reported adverse events, side effects and any occurrence of IPV or alcohol use will also be documented.

#### Study retention

We will ask participants to share their phone numbers as well as the contact of someone clost to them. Study staff will call and SMS participants in both study arms to participate in their study visits. We will conduct home visits if they miss > 1 visit.

#### Concomitant care

all antental and postnatal care is permited during the trial. Participation in other HIV trials is prohibited.

#### Outcomes

The primary outcome for Step 1 is early PrEP continuation and persistence per urine TFV at 6 months postpartum (verified post hoc via DBS analysis of TFV-DP - high persistence is defined as having levels above 2 pills per day or = > 650 fmol/punch in pregnant women and = > 950 fmol/punch in postpartum women) [31]. Secondary outcomes include:

1. **Perfect PrEP persistence:** we will measure perfect PrEP persistence (e.g., level of objective PrEP persistence measured with DBS of TFV-DP through peripartum and postpartum period at 6 and 12 months following PrEP start) as well as “prevention-effective” [32, 33] adherence among participants, which measures PrEP use prior to (7+ days) and during times of sexual activity (using self-reported recent sexual activity). We will use benchmarked blood levels from IMPAACT 2009 results for pregnant and postpartum women as= > 650 fmol/punch in pregnant women and = > 950 fmol/punch in postpartum women [31].
2. **HIV incidence** in participants (measured at each follow-up survey using SOC HIV tests)
3. **Uptake of male partner HIV testing**, measured as the proportion of male partners who test (either through HIVST or standard facility-based testing), as reported by the female participant
4. Number of participants reporting adverse events (i.e., side effects, IPV, end of relationship)

#### Step 2 (Table 2)

Differentiated PrEP distribution strategies are needed to optimize PrEP, especially in postpartum women who struggle to stay engaged in care. Similar to community-based ART delivery, we will test a differentiated PrEP delivery model for options for community PrEP delivery and pick up for postpartum women who want to continue on PrEP, but either discontinue, or have poor persistence in Step 1.

**Table 2:** Study and clinic visits by intervention and standard of care arms in Step 2 of RCT in SCOPE-PP study, Cape Town, South Africa

*If PrEP discontinued, missed visit, or poor persistence (no TFV in urine) offer new randomization (RCT 2) with chance of differentiated PrEP delivery*			
	Counseling group	Community delivery group	Steps
**Baseline study visit**	In clinic: PrEP refill and counseling with urine testing and feedback	At community pick up points^a^: PrEP refill + HIV self testing for monitoring HIV status	In study site (behind clinic): • Consent to new randomization • Survey • Reimbursement
**3 month Service visit**	In clinic: PrEP refill and counseling with urine testing and feedback	At community pick up points^a^: PrEP refill + HIV self testing for monitoring HIV status	Standard monitoring • No reimbursement for time
**6 month Service visit**	In clinic: PrEP refill and counseling with urine testing and feedback	In community^a^: PrEP refill + HIV self testing for monitoring HIV status	Standard monitoring No reimbursement for time
**9 month Study visit**	In clinic: PrEP refill and counseling with urine testing and feedback	In community^a^: PrEP refill + HIV self testing for monitoring HIV status Referral to continue on PrEP at local clinic	In study site: • Blood collection • Survey • Reimbursement for time
**End of study and referral for ongoing PrEP care in clinic**			

^a^Study will offer community pick up in local church or hall, depending on participant preference

*PrEP* pre-exposure prophylaxis, *HIVST* HIV self-testing, *TFV* tenofovir diphosphate

Participants in the intervention arm who are adherent and continued on PrEP at 6 months will continue with first randomization of sustained biofeedback through to 12-months follow-up. Participants in the SOC arm who are adherent and continued on PrEP at 6 months will continue with first randomization of SOC through 12-months follow up in clinic. Meanwhile, participants who are non-adherent (per TFV urine testing) and/or discontinued PrEP at 6 months in the Step 1 study, and want to continue in the study, will be re-randomized to Step 2.

##### Recruitment and enrollment

Postpartum women who were on PrEP in Step 1 but discontinued PrEP or were not adherent to PrEP during periods of sexual activity (prevention-effective persistence) and want to continue on PrEP will receive a second randomization to receive additional services. Participants who are eligible for Step 2 will complete a second written informed consent immediately after being screened. The inclusion criteria for Step 2 are:

- Enrolled in Step 1 of SCOPE-PP
- Discontinued PrEP (did not return for PrEP refill prescription) or were not adherent (negative urine TFV result, indicating did not take PrEP in last 48–72 hours)
- Want to continue taking PrEP
- Documented HIV-negative at the time of Step 2 screening
- Still lives within 20 km of the study facility
- Without psychiatric or medical contraindications to PrEP use
- Postpartum with live infant,
- Able and willing to consent to study participation.

Individuals not meeting the above criteria will be excluded.

##### Randomization

For Step 2, individual women will be randomized using a 1:1 ratio to either 1) persistence counseling through biofeedback and PrEP rapid collection or 2) persistence counseling, PrEP rapid collection, plus differentiated PrEP delivery (community PrEP delivery). We will use the same randomization strategy as described under Step 1.

- Intervention: Enhanced adherence counseling through biofeedback and rapid PrEP collection following HIVST

##### Intervention

We will randomize ~ 325 postpartum women from Step 1 who disengaged from PrEP during the first 6-months post enrollment and desire to stay on PrEP to:

- Intervention 1: Rapid PrEP collection following HIVST and differentiated model of community-based PrEP delivery.
- Intervention 2: Enhanced adherence counseling through biofeedback in the clinic and rapid PrEP collection following HIVST

**Community PrEP delivery** with HIVST and counselling at a community location (hall, library or NGO/church) will be available for women randomized to intervention 1 at a select, pre-determined date of the month. The counsellor will be available to answer questions and collect data on adherence and side effects. If randomized to differentiated care the participant will not receive enhanced biofeedback in the clinic (Intervention 2).

We will continue to assess study outcomes at study visits in the clinic on site including PrEP continuation and persistence and DBS to assess objective persistence. A summary of the Step 1 and Step 2 study design can be found in Table 3.

**Table 3:** SCOPE-PP Step 1 and Step 2 Study populations, Intervention, Outcomes and Hypotheses

Study	Study Population	Intervention	Outcome	Hypothesis
Step 1:	*N* = 650 **pregnant** women at enrollment	Persistence biofeedback	PrEP continuation and persistence (urine TFV at 6 months postpartum & verified post hoc via DBS of TFV-DP)	Persistence biofeedback will improve PrEP continuation & persistence by > 15% compared to standard of care (no intervention) in pregnant and early postpartum women
Step 2^a^:	*N* = Approximately 325 **postpartum** women at enrollment Composed of ALL women who want to use PrEP but are struggling to engage w/PrEP from Step 1 (EARLY-PREP-P)	Differentiated PrEP delivery (in community pick up points) with HIVST (for participant and partner) Vs. Persistence biofeedback	PrEP continuation and persistence through 12-months follow up per urine TFV (post hoc DBS analysis)	In postpartum women who want to use PrEP but struggle to engage with initial use, PrEP use continues and improves by > 15% differentiated PrEP delivery (in community pick up points) at longer time periods (through 12-months follow up)

^a^Step 2 sample size is approximate because the participants will be recruited from the Step 1 and that number will depend on how many want to be in the study and how many discontinue PrEP and/or have poor persistence per urine TFV testing

### Data collection

Participants who enroll in Step 2 will complete another survey immediately following enrollment. This survey will assess prior PrEP persistence (barriers and facilitators), HIV risk (including sexual activity, partner HIV status and risk perception), prior use of PrEP or attitudes about PrEP, and information on IPV and substance use. All surveys will be conducted in the local language by trained study staff using electronic tablets with REDCap software.

### Data safety and monitoring board

In support of the study team, a Data Safety and Monitoring Board (DSMB) comprised of senior South African and US scientists and experts will provide technical inputs to specific aspects of the study. The Chair of the DSMB will be Professor James McIntyre, Ob-Gyn, is the CEO of the Anova Health Institute and Honorary Professor in the School of Public Health & Family Medicine at UCT has significant experience in research on PMTCT and PrEP delivery in SA. Dr. McIntyre has extensive experience in chairing clinical trial DSMBs. Dr. Hasina Subedar (SA National Department of Health, PrEP lead) is the lead on PrEP roll out and guidelines in South Africa. Professor Linda-Gail Bekker (UCT, Desmond Tutu Health Foundation) is a world-renowned clinician, researcher and expert on PrEP and PrEP in adolescences and pregnant women in SA. In addition we will convene a local Community Advisory Board (CAB) in Gugulethu at the beginning of the study with meetings every 6 months to share the study design, study findings and learn more about the community perceptions, barriers and facilitators to maternal PrEP.

### Follow-up data

Similar to Step 1, participants will complete a follow-up survey every 3 months (4 follow-up surveys total). Surveys will ask about HIV risk, sexual activity and PrEP persistence, and perceptions about differentiated care (if received). Any adverse events, side effects and IPV will be recorded at each visit. Participants who report substance use, violence and/or IPV will be referred to a local NGO for social services and counseling.

### Outcomes

The primary outcome for Step 2 is continued PrEP use and persistence in later postpartum. PrEP continuation and persistence 6 months after the second randomization, (~ 12-months follow up) per urine TFV (post hoc DBS analysis) comparing enhanced counseling alone to the SOC arm. Secondary outcomes are the same as Step 1 and include: perfect PrEP persistence during Step 2, HIV incidence during Step 2, and adverse events at the same time.

### Cost-effectiveness

The conduct and reporting of the cost-effectiveness analyses will follow Consolidated Health Economic Evaluation Reporting Standards (CHEERS). All costs will be expressed according to a single price year and will be converted to US$. Costs and outcomes will be discounted at 3%, with variation in sensitivity analysis. Our study team will estimate the cost per participant in SCOPE-PP versus the standard of care from baseline to 6, 12 and 15 months postpartum and relate these costs to primary outcomes in order to estimate incremental cost-effectiveness ratios (ICERs) in natural units. For this analysis, the scope of costs will include the costs to the health system of facility-based PrEP prescription, HIV counseling and testing as well as the costs of providing maternal PrEP in the community (Table 4).

**Table 4:** SCOPE-PP Outcomes, Measures, Data Sources, and Timing (Aims 1 and 2)

Outcomes	Measures	Data Sources	Follow-up timing
**Aim 1: Efficacy of SCOPE-PP (*Primary Outcomes)***			
Efficacy of SCOPE-PP	**Perfect Persistence:** Persistence on PrEP (%, time on PrEP adherent). Perfect persistence in postpartum = > 950 fmol/punch in DBS TFV-DP), by study arm	DBS TDF-DP	RCT 1: 6 months RCT 2: 15 months postpartum
	**Prevention-Effective Persistence:** Persistence on PrEP when sexually active (in postpartum women = > 950 fmol/punch in DBS TFV-DP), by study arm	DBS TDF-DP	RCT 1: 6 months RCT 2: 15 months postpartum
	**Self-reported persistence:** self-reported measures of PrEP use and pill counts over the phone	Self-reported PrEP persistence &pill count	Quarterly
	**Secondary outcomes**: HIV incidence (maternal and infant), adverse events and IPV	RedCap clinical data	Monthly
**Nested implementation science evaluation of SCOPE-PP acceptability, feasibility using CFIR**			
Ickovic’ and Meisler’s Conceptual Factors	Evaluate factors associated with PrEP persistence in conceptual model: individual level (including partner PrEP support), facility level, HIV level (partner HIV testing & serostatus)	Quantitative surveys at each visit	Baseline, 1 month, 6 months, 12 months postpartum
Acceptability and uptake of intervention	Organizational, providers, woman and partner views on acceptability of SCOPE-PP including HIVST, urine TFV testing for persistence monitoring, and differentiated care	Mixed methods	At 6 months & 12 months follow-up of all participants (survey); IDIs in 20 providers working with PrEP, 30 participants & 30 partners
Feasibility of intervention	Organizational, providers, woman and partner views on feasibility & scalability of implementation of SCOPE-PP	Mixed methods surveys	
**Aim 2: Evaluate cost effectiveness of SCOPE-PP**			
Incremental cost of Scope-PP	Based on micro-costing, evaluate incremental cost per participant of SCOPE-PP from baseline to 6, 12 and 15 months postpartum by trial step	Quantitative surveys at each visit	RCT 1: 6 months RCT 2: 15 months postpartum
Incremental cost per improved PrEP continuation and persistence	Using trial-based cost-effectiveness analysis, evaluate incremental cost per improved PrEP continuation and persistence at 6, 12 and 15 months postpartum for SCOPE-PP (integrating step 1 and step 2 trials)	Quantitative surveys at each visit	RCT 1: 6 months RCT 2: 15 months postpartum
Incremental cost per DALY averted	Using model-based cost-utility analysis, evaluate incremental lifetime cost and DALYs averted for SCOPE-PP and assess value for money	N/A	N/A (model-based analysis)

*SCOPE-PP* Stepped care to optimize pre-exposure prophylaxis (PrEP) effectiveness in pregnant and postpartum women, *DALY* disability adjusted life year, *CFIR* Consolidated Framework for Implementation Research, *PrEP* pre-exposure prophylaxis, *HIVST* HIV self-testing, *TFV-DP* tenofovir diphosphate

### Nested implementation science data

SCOPE-PP outcomes, measures, data sources, and timing are summarized in Table 4. In addition to the surveys, we will conduct qualitative interviews with participants, healthcare workers and stakeholders as described below.

#### Acceptability and feasibility of SCOPE-PP – female participants

Surveys with female participants will be used to assess acceptability of PrEP, HIVST and urine TFV testing to increase maternal PrEP use. Acceptability is defined as the perception among women that a given intervention is agreeable, palatable, or satisfactory. Feasibility is defined as the extent to which a new intervention can be successfully used or carried out within a given organization or setting including in relation to patients of differing SES [34].

#### Qualitative methods with women

We will use qualitative methods to capture participants’ lived experiences within their social contexts [35]. Twelve months after enrollment we will sample 30 participants for semi-structured, in-depth interviews. Purposive sampling will be used to help match the characteristics of the study population. Interviews will focus on women’s attitudes about SCOPE-PP interventions including HIVST of self and partner(s), urine testing for recent persistence measurement and option for PrEP delivery for women with poor PrEP continuation and/or persistence (Aim 1).

#### Qualitative methods with male partners

In the intervention arm, we will explore men’s experiences of HIVST, barriers and facilitators and perceptions around maternal PrEP use. Collecting insights from various categories of respondents will allow us deeper understandings of the contexts and challenges involved in SCOPE-PP stepped care implementation, including those in the health system and social landscape. Interviews will cease when saturation of data is reached. Interviews will be digitally recorded, transcribed verbatim and translated.

#### Health provider interviews

Trained interviewers will conduct mixed methods interviews with 20 health care providers (nurses, managers, HIV counselors) who are actively involved in maternal PrEP services in order to assess feasibility of SCOPE-PP including maternal PrEP delivery, counselling, HIVST, PrEP counselling and rapid PrEP delivery, community PrEP delivery and urine TFV testing to improve maternal PrEP use. Data will be collected 12-months after the study enrolment begins in order to assess providers’ real-life experiences with SCOPE-PP vs standard of care (following national PrEP, HIV testing and PMTCT guidelines).

#### Key informant interviews

During study set up, we will interview 10 key informants with experience in managing the clinic (*n* = 5) and making decisions about health policy from the DOH (n = 5) to better understand key facilitators and barriers to maternal PrEP implementation (see Consolidated Framework for Implementation Research [CFIR] constructs in Table 5).

**Table 5:** Consolidated Framework for Implementation Research (CFIR) domains and constructs for SCOPE-PP

Domain (description)	Construct (description)	Maternal PrEP specific themes	Participant type
**Intervention characteristics**: Key attributes of interventions influence the success of implementation	**Relative advantage:** Stakeholders’ perception of the advantage of implementing the intervention vs standard of care	Advantage to integration PrEP in ANC services. Advantage of providing self and partner HIVST vs. facility testing, advantage of persistence counseling	Providers, women and partners
	**Complexity**: Perceived difficulty of implementation, reflected by duration, scope, radicalness, disruptiveness, centrality, and intricacy and number of steps required to implement	I am confident that I or my colleague in the clinic can integrate PrEP and HIVST into ante- and postnatal care & I am confident that I or my colleagues can follow up on PrEP in pregnant/postpartum women	Providers
	**Cost**: Costs of intervention and costs associated with implementation including supply, and opportunity costs	Concerns about cost of integration of HIVST (for women & partners) and PrEP into care	Managers and providers
**Outer Setting:** Outer context, factors external to the organization that may influence implementation	**Patient needs and resources:** The extent to which patient needs as well as barriers and facilitators to meet those needs are accurately known and prioritized by the organization	Maternal PrEP & HIVST are compatible with the needs of patients at my clinic and outcomes are achievable irrespective of patient SES	Managers, providers, women and partners
	**External Policies & Incentives:** External strategies to spread interventions including policy and regulations, external mandates, recommendations, and guidelines	Existing guidelines/policies on maternal PrEP and HIVST	Managers and providers
**Inner setting:** Inner context, factors internal to the organization that may influence implementation	**Implementation climate:** The absorptive capacity for change, and the extent to which use of that intervention will be rewarded, supported, and expected within their clinic	Leadership values evidence-based HIV practices such as maternal PrEP and HIVST	Managers and providers
	**Readiness for implementation**: Tangible and immediate indicators of organizational commitment to its decision to implement an intervention	Maternal PrEP and HIVST for patient and provider are essential parts of HIV prevention of my PMTCT program	Managers and providers
**Characteristics of Individuals:** Individuals in the organizations involved in the implementation of the intervention	**Knowledge:** Individuals’ beliefs and value placed on the intervention as well as familiarity with facts, truths, and principles related to the intervention	Awareness of DoH guidelines/policies on maternal PrEP and HIVST & Users’ skilled and provision of PrEP, counseling and HIVST	Managers and providers, women and partners
	**Attitudes:** Individuals’ attitudes toward the intervention	It is more suitable to provide maternal PrEP, HIVST and PrEP to PBFW	Managers and providers

*RCT* randomized control trial, *SCOPE-PP* Stepped care to optimize PrEP in pregnancy and postpartum, *CFIR* Consolidated Framework for Implementation Research, *PrEP* pre-exposure prophylaxis, *HIVST* HIV self-testing, *TFV-DP* tenofovir diphosphate

### Sample size considerations

Based on our PrEP-PP study which observed a mean PrEP continuation and persistence of 30% in postpartum women at 6 months, we expect an improved persistence of > 45% (mean difference of 15%) in the intervention arm of the study. This translates to the mean difference in proportion of women achieving DBS TFV-DP of > 650 fmol/punch in pregnancy and = > 950 in postpartum periods [36] by study arm. This improved PrEP persistence is in line with our pilot study findings of > 60% of women with TFV present in urine after intervention (vs. 17% in control; a 4-fold increase).

We will recruit and enroll 650 pregnant women on PrEP and follow them through 6 months in Step 1. Considering censorship due to pregnancy loss, *n* = 618 will give the study 85% power to detect a 10% + absolute difference in proportions adherent on PrEP (based on DBS of TFV-DP in postpartum period) at a two-sided significance level of 5% (Table 5).

For Step 2, we estimate that after loss to follow-up, censorship and evaluation of the interest of women to continue in the second RCT, we will follow approximately ~ 320 postpartum women who discontinued PrEP in Step 1. This will give us 80% power to detect a difference of 20% in persistence by study arm based on DBS TFV-DP levels.

### Data management

Trained study staff will capture data electronically via a secure data software, RedCap, that will include range checks and rules to promote dat quality. The trained data manager will monitor entry for completeness and consistency. They will provide the team with weekly and monthly reports to correct any missing, incomplete or inconsistent data.

### Data analysis

We will use basic measures (proportions, means with standard deviations, medians with interquartile ranges, as appropriate) and graphical displays to describe distributions of key variables. Bivariable analyses will use student’s t-tests (replaced by Mann-Whitney U-tests for non-normal distributions) or chi-square tests (replaced by exact tests for sparse data), as appropriate. Randomized arms will be compared using descriptive statistics without tests of statistical significance. Comparisons of randomized groups’ primary analyses will be by intention-to-treat, with secondary analyses based on intervention uptake. All statistical tests will be 2-sided at alpha = 0.05; all effect estimates will be reported with 95% confidence intervals (CI). For both aims, we propose a priori subgroup analyses by participant age, education, marital status in the previous 3 months at baseline, and history of participant and partner HIV testing. For subgroup analyses, effect modification will be evaluated directly from the fully adjusted model on the additive scale by calculating the relative excess risk due to interaction (RERI) and on the multiplicative scale, by examining the interaction term in regression models. Data will be analyzed in Stata version 15 (Stata Corporation, College Station, Texas).

We will evaluate the efficacy of the SCOPE-PP intervention stratified by study steps.

1. For Step 1, we will evaluate the proportion of women who continued on PrEP (defined as not missing > 1 PrEP pick up) and persisted on PrEP at 6 months following PrEP start using DBS analysis (defined as postpartum TFV-DP levels = > 950 fmol/punch in DBS) at 6 months after PrEP start and 12 months if they did not get a secondary randomization (continued and were adherent on PrEP at 6 m).
2. For Step 2, we will evaluate the proportion of women who continued on PrEP (defined as not missing > 1 PrEP pick up) and persisted on PrEP at 6 months following PrEP start using DBS analysis (defined as postpartum TFV-DP levels = > 950 fmol/punch in DBS) months at 6 months following secondary randomization (~ 12 months total) in women who had poor PrEP continuation and/or persistence in the first 6 months.
3. In addition, we will combine both steps for a complete study analysis for primary (6 m) and secondary (12 m) outcomes.

Specifically, in the primary analysis, we will include all women who start PrEP by arm and use product-limit methods including proportional hazards models to estimate the median time to PrEP persistence (at 6 and 12 months) and U-tests and other non-parametric regression models to compare persistence between intervention and control among women using PrEP. In a subsidiary analysis, we will use joint modelling framework^75^ to combine PrEP continuation and persistence measures into a single outcome variable. Women who decide to discontinue PrEP will be included in the analysis as non-adherers.

We will use Consolidated Framework for Implementation Research (CFIR) [37] to analyze qualitative data and structural and participant level acceptability and feasibility of the SCOPE-PP interventions. The CFIR is a conceptual framework that provides systematic assessment of multilevel implementation contexts to identify factors that may influence implementation and effectiveness. The CFIR comprises of five major domains: (1) the characteristics of intervention, (2) inner setting, (3) outer setting, (4) the characteristics of individuals involved, and (5) the process by which implementation is accomplished and 39 constructs that influence the implementation of evidenced-based interventions [38]. We selected 4 CFIR domains and 9 constructs per domain that were most relevant to male partner HIV testing and maternal PrEP in Table 5**.**

## Discussion

PrEP is scaling up among PBFW in sub-Saharan Africa with notable implementation successes in Kenya and ongoing demonstration projects in South Africa, Lesotho, Malawi, Zambia and Zimbabwe [19, 24, 25, 39–41] However, persistence to daily oral PrEP is challenging and data suggest that the transitional periods from pregnancy to postpartum present unique PrEP persistence barriers [19, 23, 42]. The SCOPE-PP trial will test innovative PrEP service deliver strategies specifically for postpartum women, and will step up interventions for women who are not adherent or have difficulty returning for clinical visits. To our knowledge, PrEP services have yet to distribute PrEP outside of the clinic setting (i.e., community distribution) in SA. Evidence is needed to motivate policy change for this opportunity.

In addition to behavioral and structural interventions in this study, there is a need for novel, longer-acting tools that prevent HIV acquisition in all populations, including PBFW [43, 44]. Novel PrEP agents in the pipeline are promising and will increase HIV prevention options with anticipated improved persistence and protective coverage, including dapivirine vaginal rings (DPV-VR) [45], long acting injectables [46], and newer oral ARVs developed for treatment now utilized as PrEP [47]. When longer-acting methods become available in South Africa among PBFW, we will seek to include the choice of oral-TDF PrEP and other methods in the study to align with national and international norms. We will also evaluate the cost-effectiveness in terms of cases of HIV and DALYs averted through micro-costing to inform policy and maternal PrEP scale up.

### Dissemination policy

We will work closely with the South African Department of Health and implementing partners including those in the Western Cape Province, the National DOH, and District Medical Teams (see letters of support from the National and Cape Town DOH), to develop a sustainability and dissemination plan so that, if effective and cost effective, SCOPE-PP interventions and maternal PrEP services can be more effectively and sustainably integrated into regular clinic activities. Study procedures will be developed with an eye toward sustainability (e.g., training of trainer’s strategies, development of SC fidelity tools compatible with existing tools). The dissemination plan will include stakeholder meetings and presentations to facilities, District Management Teams, and the Provincial and National DOH. Our study will help the DOH update guidelines and targets for PrEP in pregnant and breastfeeding women. Our research will be rapidly disseminated to inform policy changes, future interventions, and NIH awards, in a timely and effective manner. We will publish five or more manuscripts on the outcomes of our research in high impact journals.

## Conclusion

The SCOPE-PP trial will evaluate the impact of interventions to improve daily oral PrEP use in peripartum and postpartum women at high risk of HIV acquisition. By identifying the optimal strategy for delivering PrEP to pregnant women, this study has the potential to lead to improved HIV prevention interventions to protect against HIV acquisition and vertical transmission in women not living with HIV and their infants. This study will estimate the incremental cost effectiveness and equity impact of the intervention to inform the scale up in South Africa and throughout the region.

## Data Availability

All data will be made available through contact with the study PI at dvora.josephdavey@mednet.ucla.edu

## Abbreviations

ANC: Antental clinic
CFIR: Consolidated Framework for Implementation Research
DALY: Disability adjusted life year
DBS: Dried blood spot
DOH: Department of Health
eMTCT: Elimination of mother to child transmission
HIV: Human immunodeficiency virus
HIVST: HIV self-test
HREC: Human research ethics committee
N/A: Not applicable
PMTCT: Prevention of mother to child transmission
PrEP: Pre-exposure prophylaxis
RCT: Randomized control trial
SCOPE-PP: Stepped care to optimize pre-exposure prophylaxis (PrEP) effectiveness in pregnant and postpartum women
SOC: Standard of care
TDF-FDC: Tenofovir/emtricitabine
TFV-DP: Tenofovir diphosphate
UCT: University of Cape Town
WHO: World Health Organization
